# Antibody-Dependent Enhancement (ADE) of infection and its possible role in the pathogenesis of influenza

**DOI:** 10.1186/1753-6561-5-S1-P62

**Published:** 2011-01-10

**Authors:** Isabelle Dutry, Hui-ling Yen, Horace Lee, Malik Peiris, Martial Jaume

**Affiliations:** 1 HKU-Pasteur Research Centre, Hong Kong, Hong Kong SAR; 2 Department of Microbiology, The University of Hong Kong, Hong Kong SAR

## 

Antibody-dependent enhancement (ADE) of viral replication has been documented for viruses, such as dengue virus, Ross river virus, other alpha and flaviviruses, HIV and also influenza virus. ADE occurs when non-neutralised virus-antibody complexes find alternative receptors and routes entry into the cell via the Fc-receptor pathway. ADE has been demonstrated predominantly in macrophages or Fc-receptor bearing cells although other types of cells have also been occasionally implicated (1,2). Thus, viruses may find routes of entry to cells lacking the usual virus receptor. Alternatively, the innate immune signals triggered by virus infection via the FcR pathway may be different to those triggered by entry via the physiological virus receptor. Both these consequences may have implications for virus tropism and pathogenesis. Recently there have been reports of increased infection rates by pandemic influenza H1N1 virus following receipt of seasonal flu vaccination (3,4). ADE has been proposed as one mechanism. Here we illustrate the possible role played by humoral immunity in providing Influenza A viruses an opportunity to better infect immune cells.

Our results showed that, in some cases, prior addition of human serum to the inoculum triggered an enhanced infection of target cells as illustrated by a ~2-5 fold increase in Influenza M-gene copy numbers. Immunofluorescent microscopy revealed that serum-mediated pdmH1N1 infection led to a higher number of infected cells. As the fold increase of infected cells paralleled the fold change in viral gene copies, we conclude that ADE was acting by increasing the number of infected cells rather than solely increasing the viral load per cell.

Based on their ability to enhance pdmH1N1 infectivity, human sera could be divided into 3 distinct groups: some showing neutralization of infection, others increasing the yield of pdmH1N1-infected cells. The third group were those with no or marginal increase of virus infection, one which was not sustained over the tested range of serum dilutions.

Our results demonstrate that the newly emerged pandemic H1N1 Influenza A virus infection of cells of the hematopoietic lineage may be enhanced by the presence of some human sera. In the light of the recent studies that report possible associations between vaccination and increased susceptibility to influenza infection, it is of relevance to deepen our understanding of the biological significance and molecular mechanisms underlying serum-mediated ADE infection of influenza virus.
